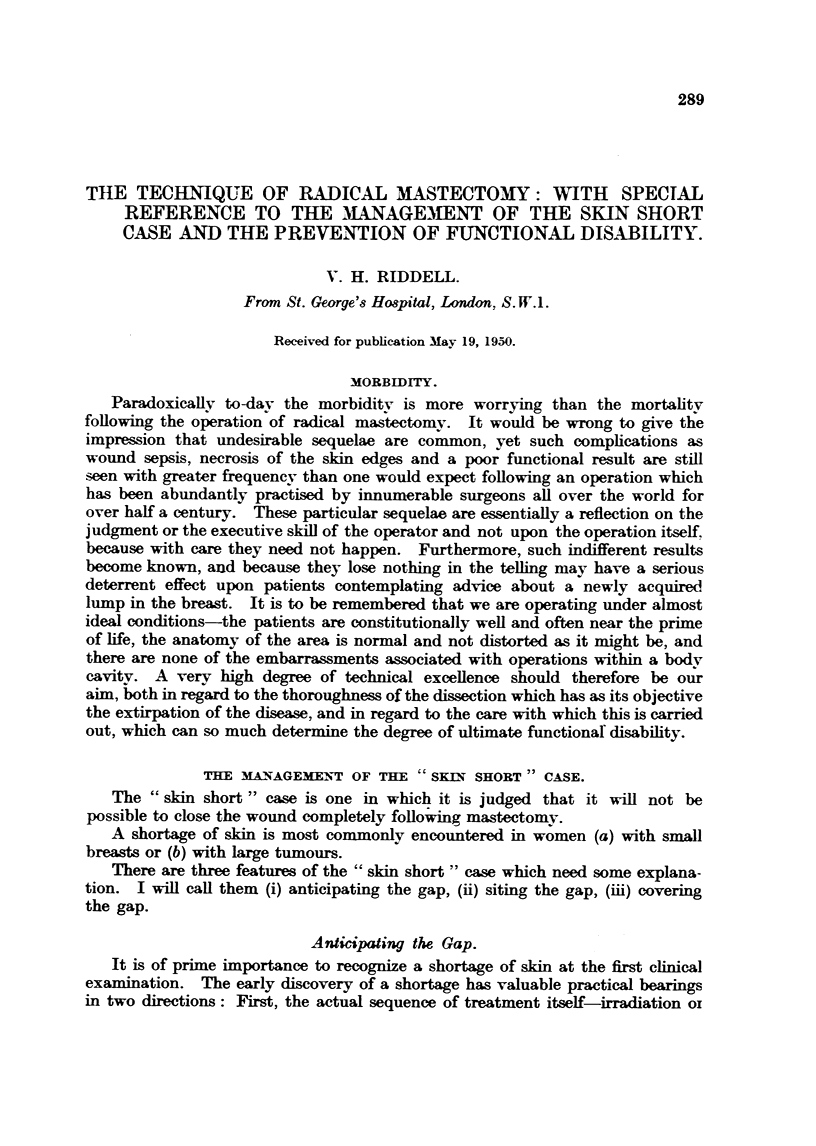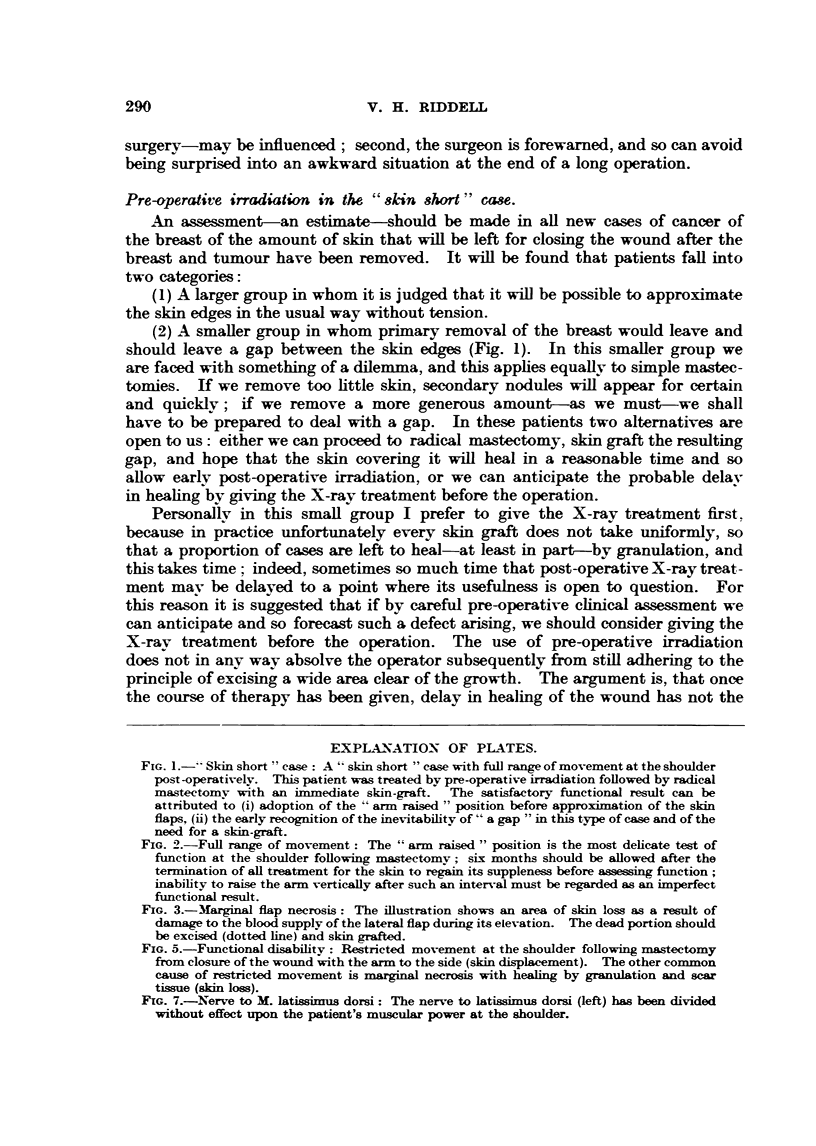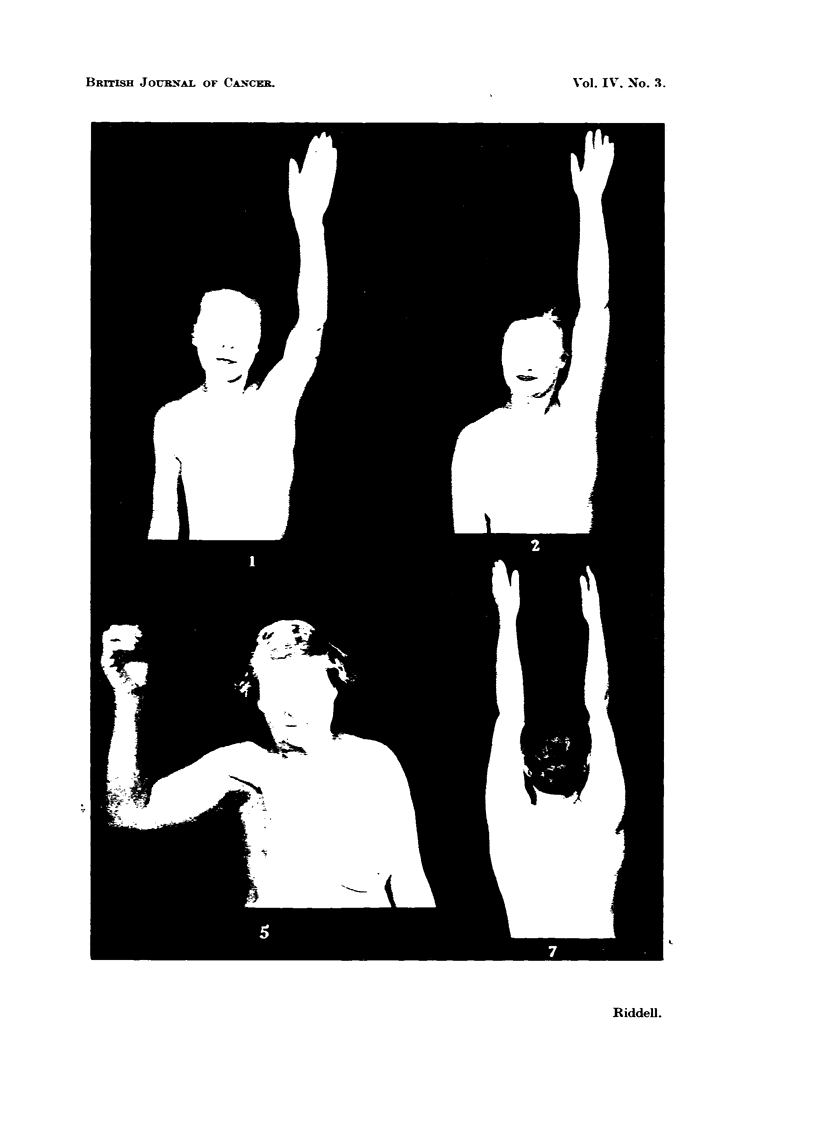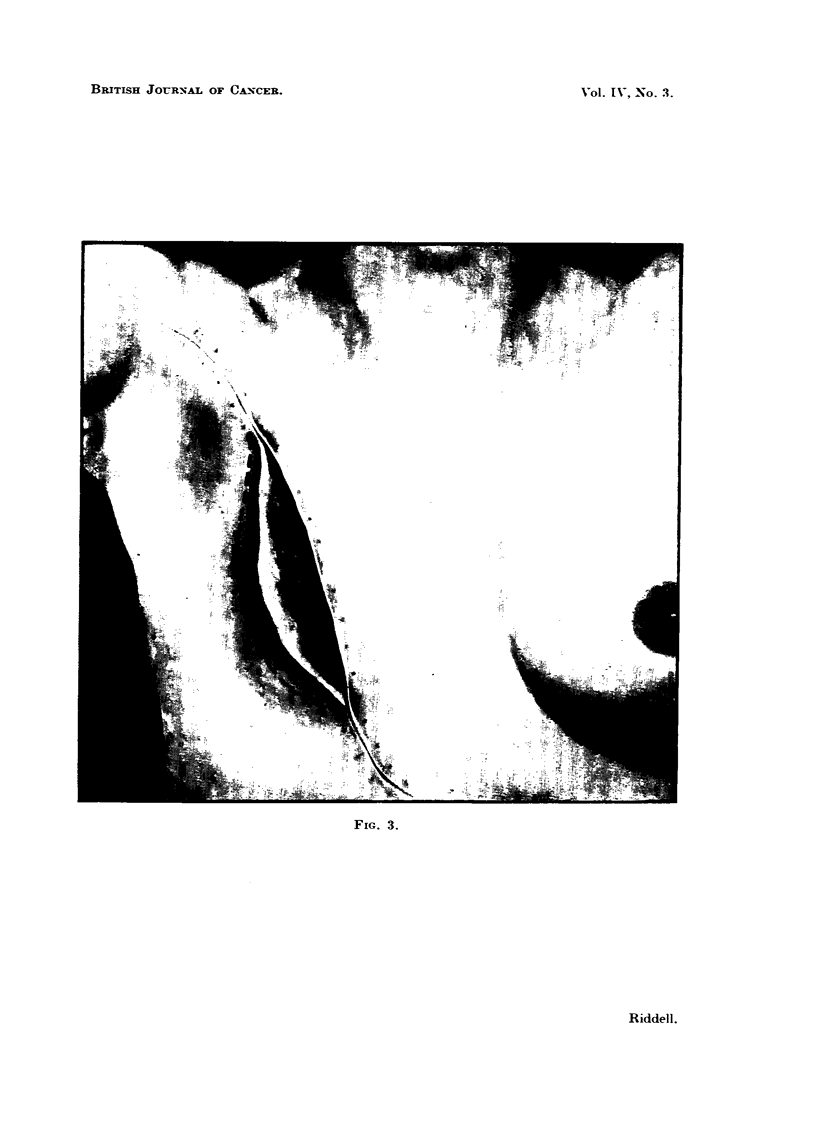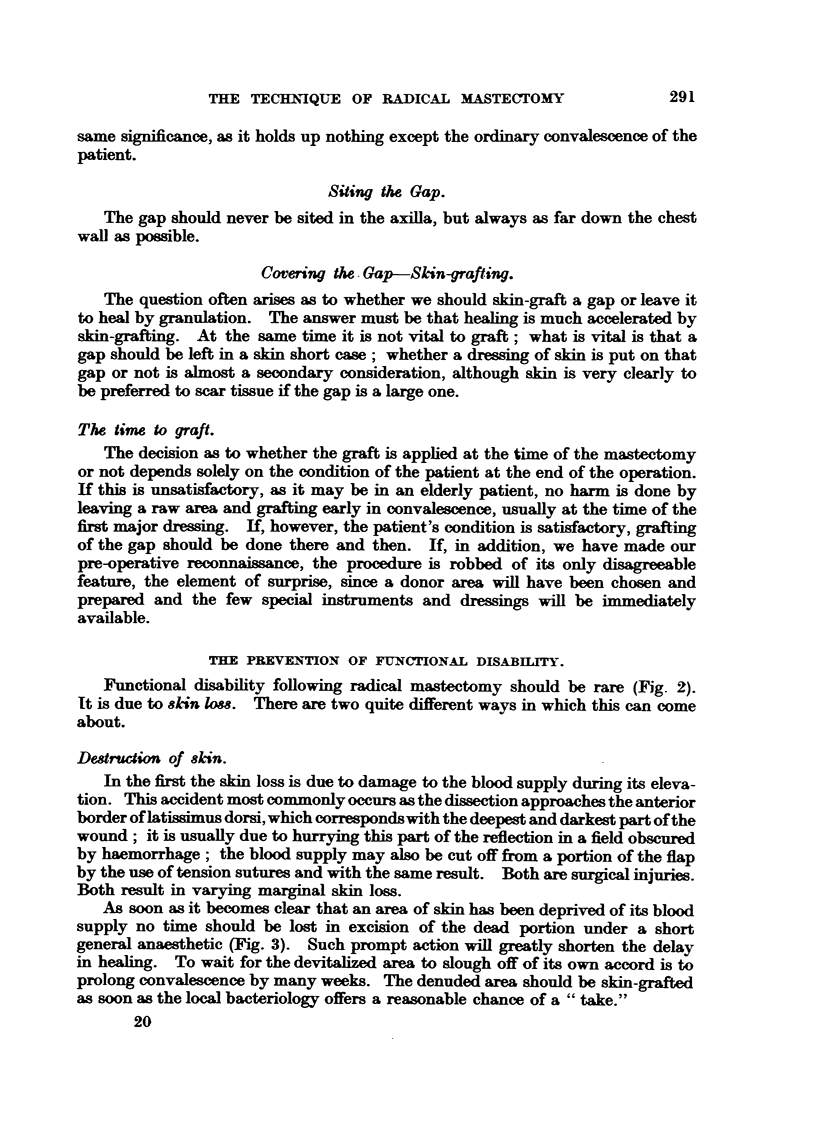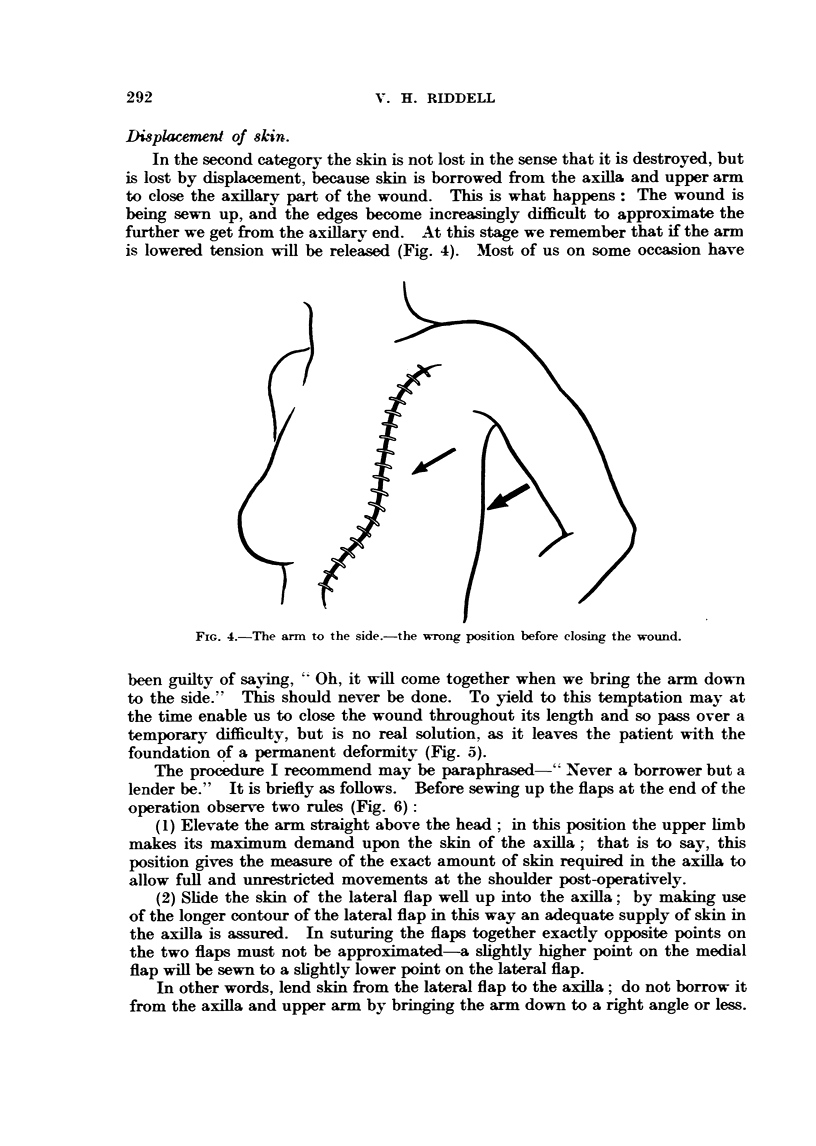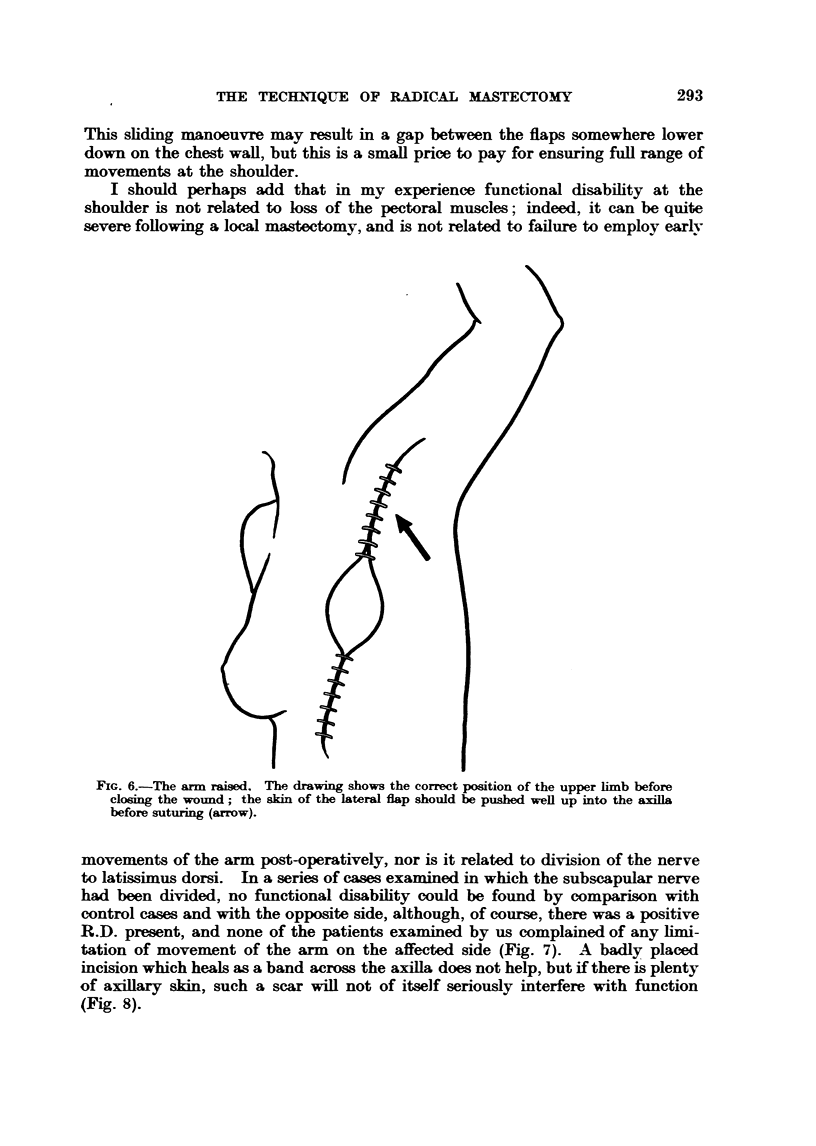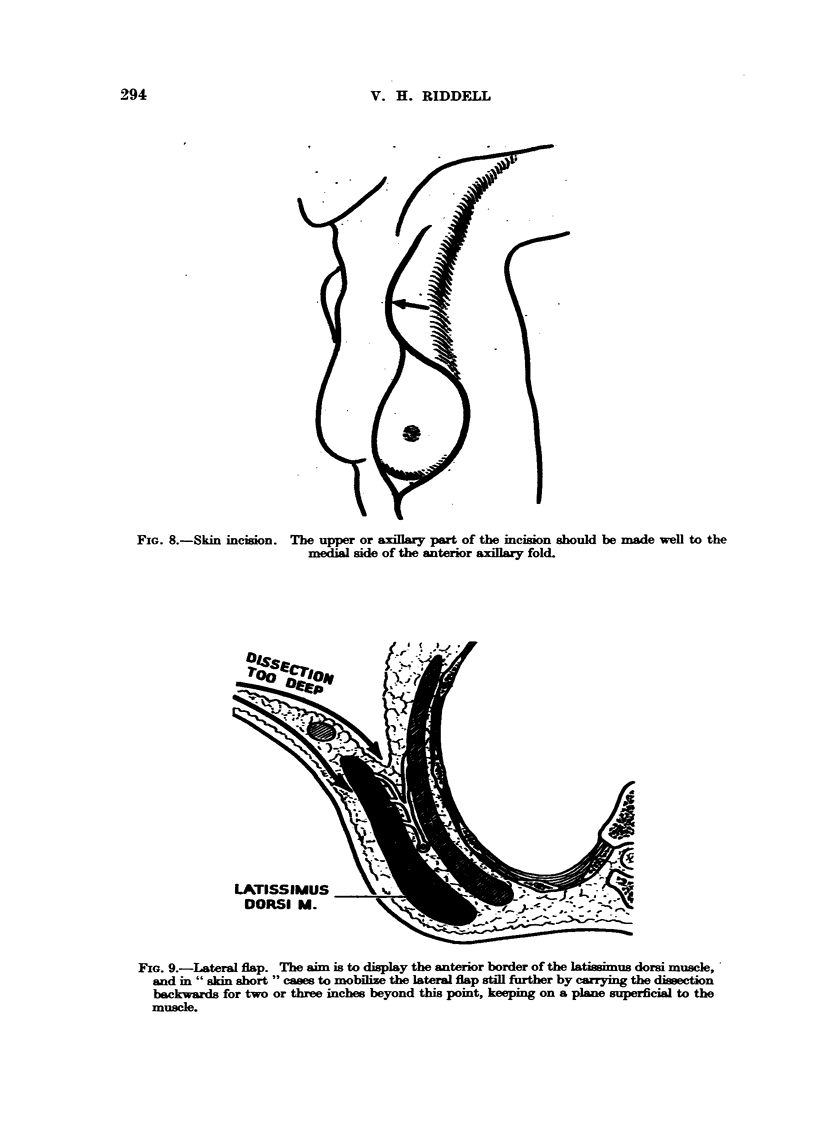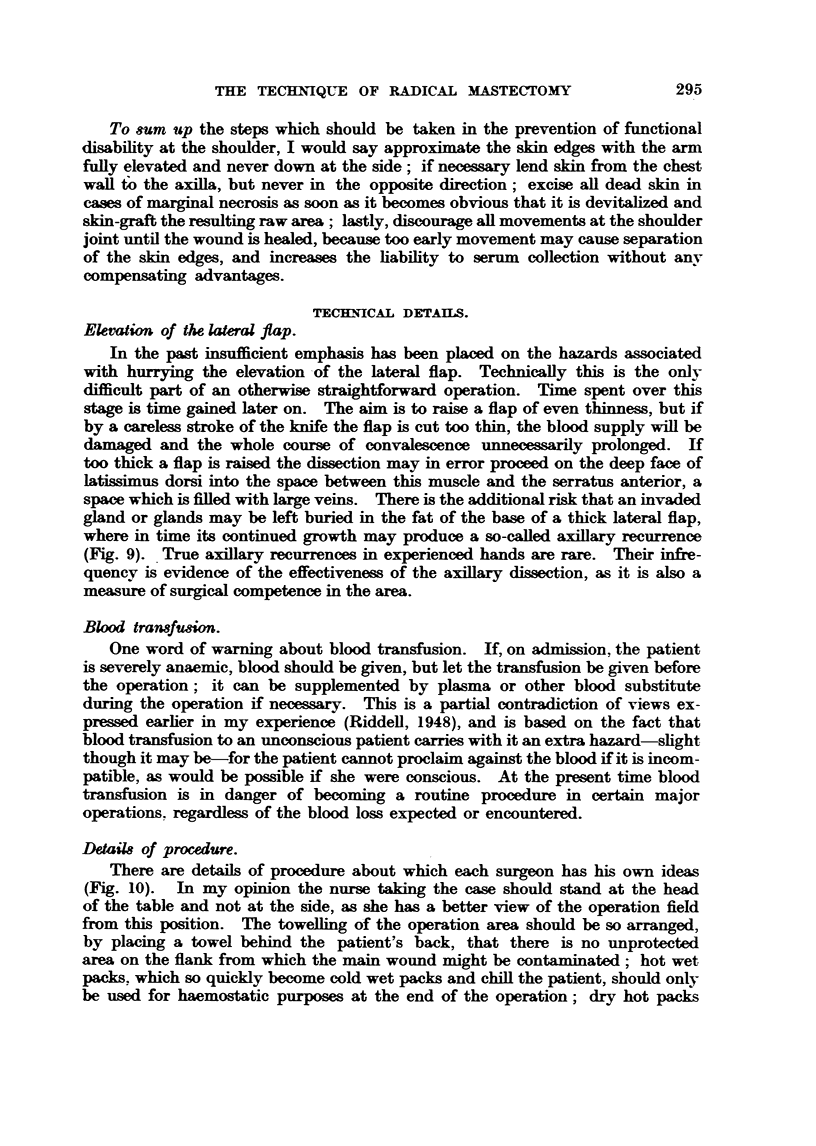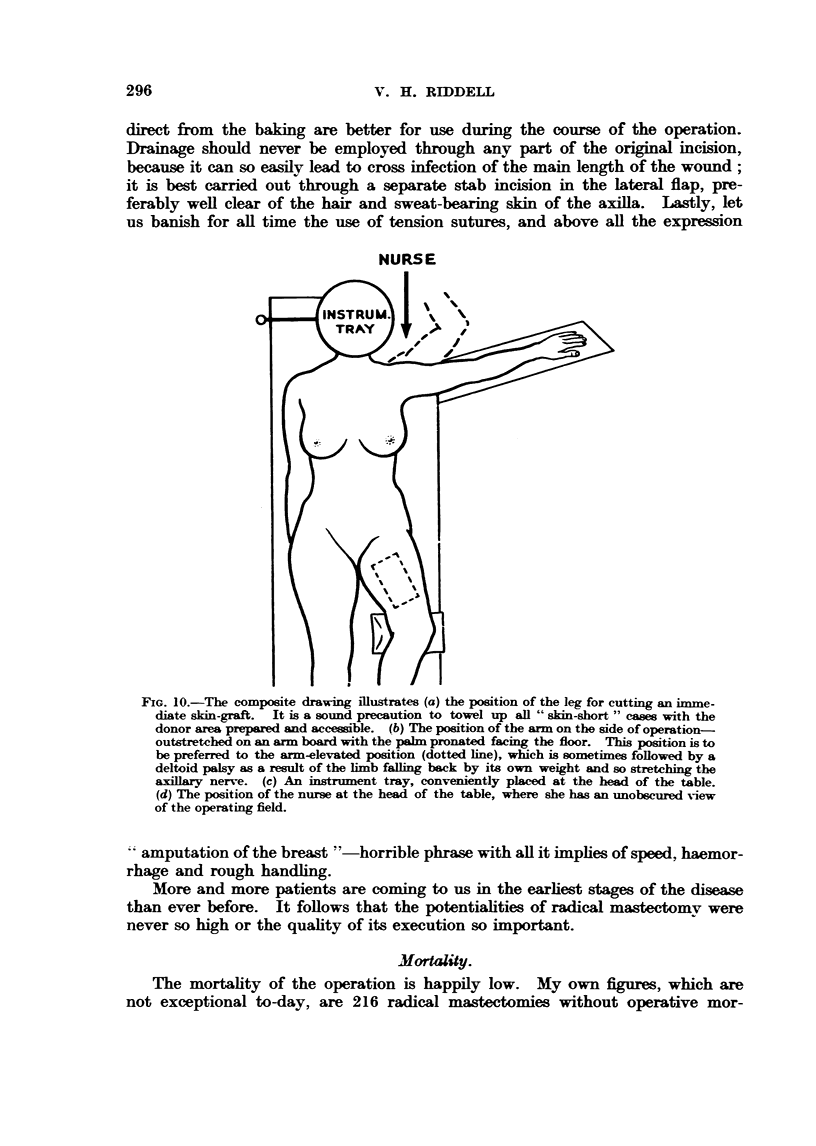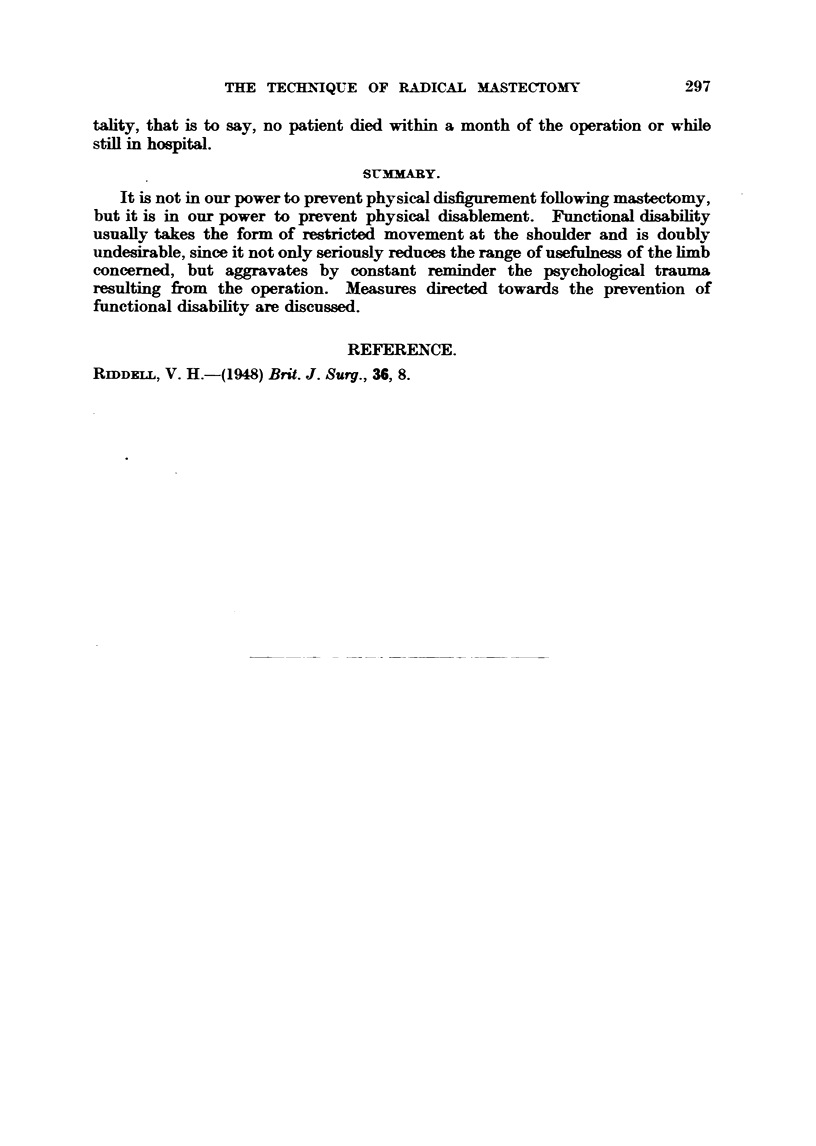# The Technique of Radical Mastectomy: With Special Reference to the Management of the Skin Short Case and the Prevention of Functional Disability

**DOI:** 10.1038/bjc.1950.27

**Published:** 1950-09

**Authors:** V. H. Riddell

## Abstract

**Images:**


### THE TECHNIQUE OF RADICAL MASTECTOMY: WITH SPECIAL REFERENCE TO THE MANAGEMENT OF THE SKIN SHORT CASE AND THE PREVENTION OF FUNCTIONAL DISABILITY.

V. H. RIDDELL.

*From St. George's Hospital, London, S.W.1.*

Received for publication May 19, 1950.

### MORBIDITY.

Paradoxically to-day the morbidity is more worrying than the mortality following the operation of radical mastectomy. It would be wrong to give the impression that undesirable sequelae are common, yet such complications as wound sepsis, necrosis of the skin edges and a poor functional result are still seen with greater frequency than one would expect following an operation which has been abundantly practised by innumerable surgeons all over the world for over half a century. These particular sequelae are essentially a reflection on the judgment or the executive skill of the operator and not upon the operation itself, because with care they need not happen. Furthermore, such indifferent results become known, and because they lose nothing in the telling may have a serious deterrent effect upon patients contemplating advice about a newly acquired lump in the breast. It is to be remembered that we are operating under almost ideal conditions—the patients are constitutionally well and often near the prime of life, the anatomy of the area is normal and not distorted as it might be, and there are none of the embarrassments associated with operations within a body cavity. A very high degree of technical excellence should therefore be our aim, both in regard to the thoroughness of the dissection which has as its objective the extirpation of the disease, and in regard to the care with which this is carried out, which can so much determine the degree of ultimate functional disability.

### THE MANAGEMENT OF THE "SKIN SHORT" CASE.

The "skin short" case is one in which it is judged that it will not be possible to close the wound completely following mastectomy.

A shortage of skin is most commonly encountered in women (*a*) with small breasts or (*b*) with large tumours.

There are three features of the "skin short" case which need some explanation. I will call them (i) anticipating the gap, (ii) siting the gap, (iii) covering the gap.

### *Anticipating the Gap.*

It is of prime importance to recognize a shortage of skin at the first clinical examination. The early discovery of a shortage has valuable practical bearings in two directions: First, the actual sequence of treatment itself—irradiation or surgery—may be influenced; second, the surgeon is forewarned, and so can avoid being surprised into an awkward situation at the end of a long operation.

*Pre-operative irradiation in the "skin short" case.*

An assessment—an estimate—should be made in all new cases of cancer of the breast of the amount of skin that will be left for closing the wound after the breast and tumour have been removed. It will be found that patients fall into two categories:

(1) A larger group in whom it is judged that it will be possible to approximate the skin edges in the usual way without tension.

(2) A smaller group in whom primary removal of the breast would leave and should leave a gap between the skin edges (Fig. 1). In this smaller group we are faced with something of a dilemma, and this applies equally to simple mastectomies. If we remove too little skin, secondary nodules will appear for certain and quickly; if we remove a more generous amount—as we must—we shall have to be prepared to deal with a gap. In these patients two alternatives are open to us: either we can proceed to radical mastectomy, skin graft the resulting gap, and hope that the skin covering it will heal in a reasonable time and so allow early post-operative irradiation, or we can anticipate the probable delay in healing by giving the X-ray treatment before the operation.

Personally in this small group I prefer to give the X-ray treatment first, because in practice unfortunately every skin graft does not take uniformly, so that a proportion of cases are left to heal—at least in part—by granulation, and this takes time; indeed, sometimes so much time that post-operative X-ray treatment may be delayed to a point where its usefulness is open to question. For this reason it is suggested that if by careful pre-operative clinical assessment we can anticipate and so forecast such a defect arising, we should consider giving the X-ray treatment before the operation. The use of pre-operative irradiation does not in any way absolve the operator subsequently from still adhering to the principle of excising a wide area clear of the growth. The argument is, that once the course of therapy has been given, delay in healing of the wound has not the

---

EXPLANATION OF PLATES.

FIG. 1.—"Skin short" case: A "skin short" case with full range of movement at the shoulder post-operatively. This patient was treated by pre-operative irradiation followed by radical mastectomy with an immediate skin-graft. The satisfactory functional result can be attributed to (i) adoption of the "arm raised" position before approximation of the skin flaps, (ii) the early recognition of the inevitability of "a gap" in this type of case and of the need for a skin-graft.

FIG. 2.—Full range of movement: The "arm raised" position is the most delicate test of function at the shoulder following mastectomy; six months should be allowed after the termination of all treatment for the skin to regain its suppleness before assessing function; inability to raise the arm vertically after such an interval must be regarded as an imperfect functional result.

FIG. 3.—Marginal flap necrosis: The illustration shows an area of skin loss as a result of damage to the blood supply of the lateral flap during its elevation. The dead portion should be excised (dotted line) and skin grafted.

FIG. 5.—Functional disability: Restricted movement at the shoulder following mastectomy from closure of the wound with the arm to the side (skin displacement). The other common cause of restricted movement is marginal necrosis with healing by granulation and scar tissue (skin loss).

FIG. 7.—Nerve to M. latissimus dorsi: The nerve to latissimus dorsi (left) has been divided without effect upon the patient's muscular power at the shoulder.

Riddell.

Fig. 3.

Riddell.

same significance, as it holds up nothing except the ordinary convalescence of the patient.

### *Siting the Gap.*

The gap should never be sited in the axilla, but always as far down the chest wall as possible.

### *Covering the Gap—Skin-grafting.*

The question often arises as to whether we should skin-graft a gap or leave it to heal by granulation. The answer must be that healing is much accelerated by skin-grafting. At the same time it is not vital to graft ; what is vital is that a gap should be left in a skin short case ; whether a dressing of skin is put on that gap or not is almost a secondary consideration, although skin is very clearly to be preferred to scar tissue if the gap is a large one.

*The time to graft.*

The decision as to whether the graft is applied at the time of the mastectomy or not depends solely on the condition of the patient at the end of the operation. If this is unsatisfactory, as it may be in an elderly patient, no harm is done by leaving a raw area and grafting early in convalescence, usually at the time of the first major dressing. If, however, the patient's condition is satisfactory, grafting of the gap should be done there and then. If, in addition, we have made our pre-operative reconnaissance, the procedure is robbed of its only disagreeable feature, the element of surprise, since a donor area will have been chosen and prepared and the few special instruments and dressings will be immediately available.

### THE PREVENTION OF FUNCTIONAL DISABILITY.

Functional disability following radical mastectomy should be rare (Fig. 2). It is due to *skin loss*. There are two quite different ways in which this can come about.

*Destruction of skin.*

In the first the skin loss is due to damage to the blood supply during its elevation. This accident most commonly occurs as the dissection approaches the anterior border of latissimus dorsi, which corresponds with the deepest and darkest part of the wound ; it is usually due to hurrying this part of the reflection in a field obscured by haemorrhage ; the blood supply may also be cut off from a portion of the flap by the use of tension sutures and with the same result. Both are surgical injuries. Both result in varying marginal skin loss.

As soon as it becomes clear that an area of skin has been deprived of its blood supply no time should be lost in excision of the dead portion under a short general anaesthetic (Fig. 3). Such prompt action will greatly shorten the delay in healing. To wait for the devitalized area to slough off of its own accord is to prolong convalescence by many weeks. The denuded area should be skin-grafted as soon as the local bacteriology offers a reasonable chance of a " take."

*Displacement of skin.*

In the second category the skin is not lost in the sense that it is destroyed, but is lost by displacement, because skin is borrowed from the axilla and upper arm to close the axillary part of the wound. This is what happens : The wound is being sewn up, and the edges become increasingly difficult to approximate the further we get from the axillary end. At this stage we remember that if the arm is lowered tension will be released (Fig. 4). Most of us on some occasion have

FIG. 4.—The arm to the side.—the wrong position before closing the wound.

been guilty of saying, " Oh, it will come together when we bring the arm down to the side." This should never be done. To yield to this temptation may at the time enable us to close the wound throughout its length and so pass over a temporary difficulty, but is no real solution, as it leaves the patient with the foundation of a permanent deformity (Fig. 5).

The procedure I recommend may be paraphrased—" Never a borrower but a lender be." It is briefly as follows. Before sewing up the flaps at the end of the operation observe two rules (Fig. 6) :

(1) Elevate the arm straight above the head ; in this position the upper limb makes its maximum demand upon the skin of the axilla ; that is to say, this position gives the measure of the exact amount of skin required in the axilla to allow full and unrestricted movements at the shoulder post-operatively.

(2) Slide the skin of the lateral flap well up into the axilla ; by making use of the longer contour of the lateral flap in this way an adequate supply of skin in the axilla is assured. In suturing the flaps together exactly opposite points on the two flaps must not be approximated—a slightly higher point on the medial flap will be sewn to a slightly lower point on the lateral flap.

In other words, lend skin from the lateral flap to the axilla ; do not borrow it from the axilla and upper arm by bringing the arm down to a right angle or less.

This sliding manoeuvre may result in a gap between the flaps somewhere lower down on the chest wall, but this is a small price to pay for ensuring full range of movements at the shoulder.

I should perhaps add that in my experience functional disability at the shoulder is not related to loss of the pectoral muscles; indeed, it can be quite severe following a local mastectomy, and is not related to failure to employ early

FIG. 6.—The arm raised. The drawing shows the correct position of the upper limb before closing the wound; the skin of the lateral flap should be pushed well up into the axilla before suturing (arrow).

movements of the arm post-operatively, nor is it related to division of the nerve to latissimus dorsi. In a series of cases examined in which the subscapular nerve had been divided, no functional disability could be found by comparison with control cases and with the opposite side, although, of course, there was a positive R.D. present, and none of the patients examined by us complained of any limitation of movement of the arm on the affected side (Fig. 7). A badly placed incision which heals as a band across the axilla does not help, but if there is plenty of axillary skin, such a scar will not of itself seriously interfere with function (Fig. 8).

Fig. 8.—Skin incision. The upper or axillary part of the incision should be made well to the medial side of the anterior axillary fold.

Fig. 9.—Lateral flap. The aim is to display the anterior border of the latissimus dorsi muscle, and in " skin short " cases to mobilize the lateral flap still further by carrying the dissection backwards for two or three inches beyond this point, keeping on a plane superficial to the muscle.

*To sum up* the steps which should be taken in the prevention of functional disability at the shoulder, I would say approximate the skin edges with the arm fully elevated and never down at the side ; if necessary lend skin from the chest wall to the axilla, but never in the opposite direction ; excise all dead skin in cases of marginal necrosis as soon as it becomes obvious that it is devitalized and skin-graft the resulting raw area ; lastly, discourage all movements at the shoulder joint until the wound is healed, because too early movement may cause separation of the skin edges, and increases the liability to serum collection without any compensating advantages.

### TECHNICAL DETAILS.

*Elevation of the lateral flap.*

In the past insufficient emphasis has been placed on the hazards associated with hurrying the elevation of the lateral flap. Technically this is the only difficult part of an otherwise straightforward operation. Time spent over this stage is time gained later on. The aim is to raise a flap of even thinness, but if by a careless stroke of the knife the flap is cut too thin, the blood supply will be damaged and the whole course of convalescence unnecessarily prolonged. If too thick a flap is raised the dissection may in error proceed on the deep face of latissimus dorsi into the space between this muscle and the serratus anterior, a space which is filled with large veins. There is the additional risk that an invaded gland or glands may be left buried in the fat of the base of a thick lateral flap, where in time its continued growth may produce a so-called axillary recurrence (Fig. 9). True axillary recurrences in experienced hands are rare. Their infrequency is evidence of the effectiveness of the axillary dissection, as it is also a measure of surgical competence in the area.

*Blood transfusion.*

One word of warning about blood transfusion. If, on admission, the patient is severely anaemic, blood should be given, but let the transfusion be given before the operation ; it can be supplemented by plasma or other blood substitute during the operation if necessary. This is a partial contradiction of views expressed earlier in my experience (Riddell, 1948), and is based on the fact that blood transfusion to an unconscious patient carries with it an extra hazard—slight though it may be—for the patient cannot proclaim against the blood if it is incompatible, as would be possible if she were conscious. At the present time blood transfusion is in danger of becoming a routine procedure in certain major operations, regardless of the blood loss expected or encountered.

*Details of procedure.*

There are details of procedure about which each surgeon has his own ideas (Fig. 10). In my opinion the nurse taking the case should stand at the head of the table and not at the side, as she has a better view of the operation field from this position. The towelling of the operation area should be so arranged, by placing a towel behind the patient's back, that there is no unprotected area on the flank from which the main wound might be contaminated ; hot wet packs, which so quickly become cold wet packs and chill the patient, should only be used for haemostatic purposes at the end of the operation ; dry hot packs direct from the baking are better for use during the course of the operation. Drainage should never be employed through any part of the original incision, because it can so easily lead to cross infection of the main length of the wound; it is best carried out through a separate stab incision in the lateral flap, preferably well clear of the hair and sweat-bearing skin of the axilla. Lastly, let us banish for all time the use of tension sutures, and above all the expression

Fig. 10.—The composite drawing illustrates (*a*) the position of the leg for cutting an immediate skin-graft. It is a sound precaution to towel up all " skin-short " cases with the donor area prepared and accessible. (*b*) The position of the arm on the side of operation—outstretched on an arm board with the palm pronated facing the floor. This position is to be preferred to the arm-elevated position (dotted line), which is sometimes followed by a deltoid palsy as a result of the limb falling back by its own weight and so stretching the axillary nerve. (*c*) An instrument tray, conveniently placed at the head of the table. (*d*) The position of the nurse at the head of the table, where she has an unobscured view of the operating field.

" amputation of the breast "—horrible phrase with all it implies of speed, haemorrhage and rough handling.

More and more patients are coming to us in the earliest stages of the disease than ever before. It follows that the potentialities of radical mastectomy were never so high or the quality of its execution so important.

### *Mortality.*

The mortality of the operation is happily low. My own figures, which are not exceptional to-day, are 216 radical mastectomies without operative mortality, that is to say, no patient died within a month of the operation or while still in hospital.

SUMMARY.

It is not in our power to prevent physical disfigurement following mastectomy, but it is in our power to prevent physical disablement. Functional disability usually takes the form of restricted movement at the shoulder and is doubly undesirable, since it not only seriously reduces the range of usefulness of the limb concerned, but aggravates by constant reminder the psychological trauma resulting from the operation. Measures directed towards the prevention of functional disability are discussed.

REFERENCE.

Riddell, V. H.—(1948) *Brit. J. Surg.*, **36**, 8.